# Improving Antimicrobial Delivery for Pediatric Patients in ED: Focus on Pharmacy Workflow

**DOI:** 10.1097/pq9.0000000000000702

**Published:** 2024-02-19

**Authors:** Susan Butler, Kimberly Nissen, Shannon Draus, Bryan Snook

**Affiliations:** From the * Geisinger Janet Weis Children’s Hospital, Danville, Pa.; † Geisinger Danville, Pa.

## Introduction:

The 2020 Surviving Sepsis Campaign pediatric guidelines provide specific recommendations for the timing of antimicrobial administration in septic patients. They recommend administering intravenous (IV) antimicrobials as soon as possible, within 1 hour of recognition for septic shock, and 3 hours for sepsis-associated organ dysfunction without shock.^1^ When this project was conducted, all pediatric patients requiring emergency care at our institution were cared for within an adult-oriented emergency department (ED). The desired time of less than 1 hour to first antimicrobial was not met for pediatric ED patients. No formal pharmacy workflow existed for handling pediatric medication orders in the ED, and inconsistencies could contribute to antimicrobial administration delays. Additionally, there needed to be clearer communication channels for addressing delays and improved coordination between pediatric pharmacy and emergency medicine providers.

## Objective:

Create and implement a pediatric-specific pharmacy workflow within an adult-focused ED to optimize efficiency and improve adherence to antimicrobial administration in the first hour of resuscitation.

## Methods:

Process mapping was completed to identify inefficiencies in the existing process. With input from key stakeholders, a pediatric-specific pharmacy workflow was created to identify the pharmacist responsible for verifying pediatric ED orders, designate the pharmacy where medications would be prepared (central inpatient pharmacy versus pediatric satellite pharmacy), incorporate scanning into the medication delivery process, and outline communication channels and expectations. The workflow was reviewed with all pharmacy staff and applicable pediatric emergency medicine staff. The pharmacy workflow for pediatric ED patients was implemented in November 2019. Data was collected pre- and postimplementation with a 1-month washout period. Orders for IV antimicrobials not stocked in the automated dispensing cabinet for patients less than 18 years of age in the ED at Geisinger Medical Center were included. Orders with a start time greater than 1 hour from when the order was entered, orders with an “on call” frequency intended for surgical prophylaxis, and orders for medications with no dose administrations documented were excluded. Summary statistics were used to compare medication process data before and after implementation.

## Results:

There were observed reductions in all areas of the process (Table 1) except for time from order placement to verification. Stat antimicrobial delivery in <30 minutes increased to 73%. The time to administration of any antimicrobial decreased by 20 minutes, and the time to first antimicrobial administration decreased by 18 minutes (20%). Adherence to first antimicrobial administration <1 hour increased from 39% to 51%.

**Table 1.** Impact of Workflow on Key Intervals of Medication Order Processing

Interval	Preimplementation Average	Postimplementation Average	Average Change (Min)	Percent Difference
Order placement to verification	5.6 min (n = 98)	7.1 min (n = 84)	+1.5 min	+26.8%
Verification to preparation	11.5 min (n = 40)	9.4 min (n = 42)	−2.1 min	−18.3%
Preparation to checking	18.5 min (n = 37)	10.4 min (n = 42)	−8.1 min	−43.8%
Checking to administration	77.5 min (n = 86)	60.0 min (n = 84)	−17.5 min	−22.6%
Order placement to administration	105 min (n = 98)	85 min (n = 84)	−20 min	−19.1%

## Conclusions:

Process mapping helped identify operational inefficiencies within the medication process. Optimizing pharmacy workflow for pediatric patients in the ED by defining responsibilities and channels for clear, direct interdisciplinary communication notably improved overall efficiency, stat antimicrobial delivery, antimicrobial administration times, and the less than 1-hour pediatric sepsis metric.
